# Author Correction: Hydrogels with tunable mechanical plasticity regulate endothelial cell outgrowth in vasculogenesis and angiogenesis

**DOI:** 10.1038/s41467-024-47722-6

**Published:** 2024-04-16

**Authors:** Zhao Wei, Meng Lei, Yaohui Wang, Yizhou Xie, Xueyong Xie, Dongwei Lan, Yuanbo Jia, Jingyi Liu, Yufei Ma, Bo Cheng, Sharon Gerecht, Feng Xu

**Affiliations:** 1 The Key Laboratory of Biomedical Information Engineering of Ministry of Education, School of Life Science and Technology, Xi’an Jiaotong University, Xi’an, 710049 PR China; 2 Bioinspired Engineering and Biomechanics Center (BEBC), Xi’an Jiaotong University, Xi’an, 710049 PR China; 3 Department of Biomedical Engineering, Duke University, Durham, NC 27708 USA

**Keywords:** Angiogenesis, Gels and hydrogels, Biopolymers in vivo

Correction to: *Nature Communications* 10.1038/s41467-023-43768-0, published online 14 December 2023

The original version of this article contained an error in < location: Pg. 5, right column>, which incorrectly reads ‘<vascular endothelial calmodulin>.’ The correct version states ‘<vascular endothelial cadherin >’ in place of ‘<vascular endothelial calmodulin >’. This has been corrected in both the PDF and HTML versions of the article.

