# Supplementary material for: Hydrogels with tunable mechanical plasticity regulate endothelial cell outgrowth in vasculogenesis and angiogenesis
Source: Nat Commun. 2023 Dec 14;14:8307. doi: 10.1038/s41467-023-43768-0 (PMC10721650; doi:10.1038/s41467-023-43768-0)
Supplement: Supplementary file 1 — Supplementary Information [file 41467_2023_43768_MOESM1_ESM.pdf]

Supplementary Information

Hydrogels with Tunable Mechanical Plasticity Regulate Endothelial Cell Outgrowth in Vasculogenesis and Angiogenesis

Zhao Wei[1,2#], Meng Lei[1,2#], Yaohui Wang[1,2], Yizhou Xie[1,2], Xueyong Xie[1,2], Dongwei Lan[1,2], Yuanbo Jia[1,2], Jingyi Liu[1,2], Yufei Ma[1,2], Bo Cheng[1,2], Sharon Gerecht[3*], Feng Xu[1,2*]

[1] *The Key Laboratory of Biomedical Information Engineering of Ministry of Education, School of Life Science and Technology, Xi'an Jiaotong University, Xi'an 710049, P.R. China.*

[2] *Bioinspired Engineering and Biomechanics Center (BEBC), Xi'an Jiaotong University, Xi'an 710049, P.R. China.*

[3] *Department of Biomedical Engineering, Duke University, Durham, NC 27708, USA.*

[#] *The authors contributed equally to this work.*

[*] *Corresponding authors: fengxu@mail.xjtu.edu.cn; sharon.gerecht@duke.edu*

Supplementary Figures

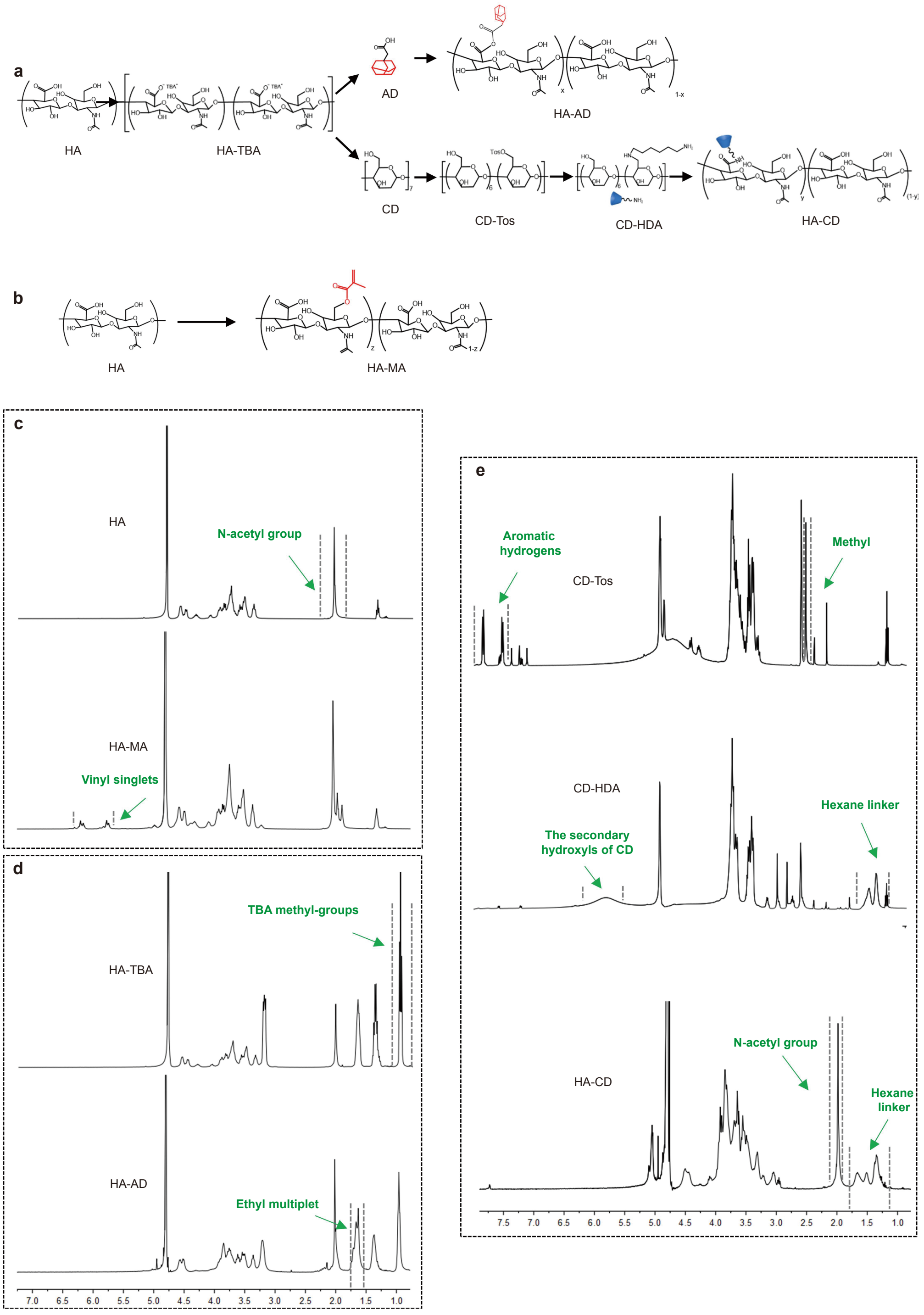

Supplementary Figure 1 | Synthesis procedures and ^{1}H NMR spectra of the different derivatives.

a-b, Synthesis routes of **(a)** HA-AD, HA-CD, and **(b)** HA-MA. **c-e,** ^{1}H NMR spectra confirm the obtained **(c)** HA-MA (solvent: D_2O), **(d)** HA-TBA (solvent: D_2O), HA-AD

(solvent: D_2O), and **(e)** CD-Tos (solvent: DMSO-d_6), CD-HAD (solvent: DMSO-d_6), HA-CD (solvent: D_2O) products, respectively.

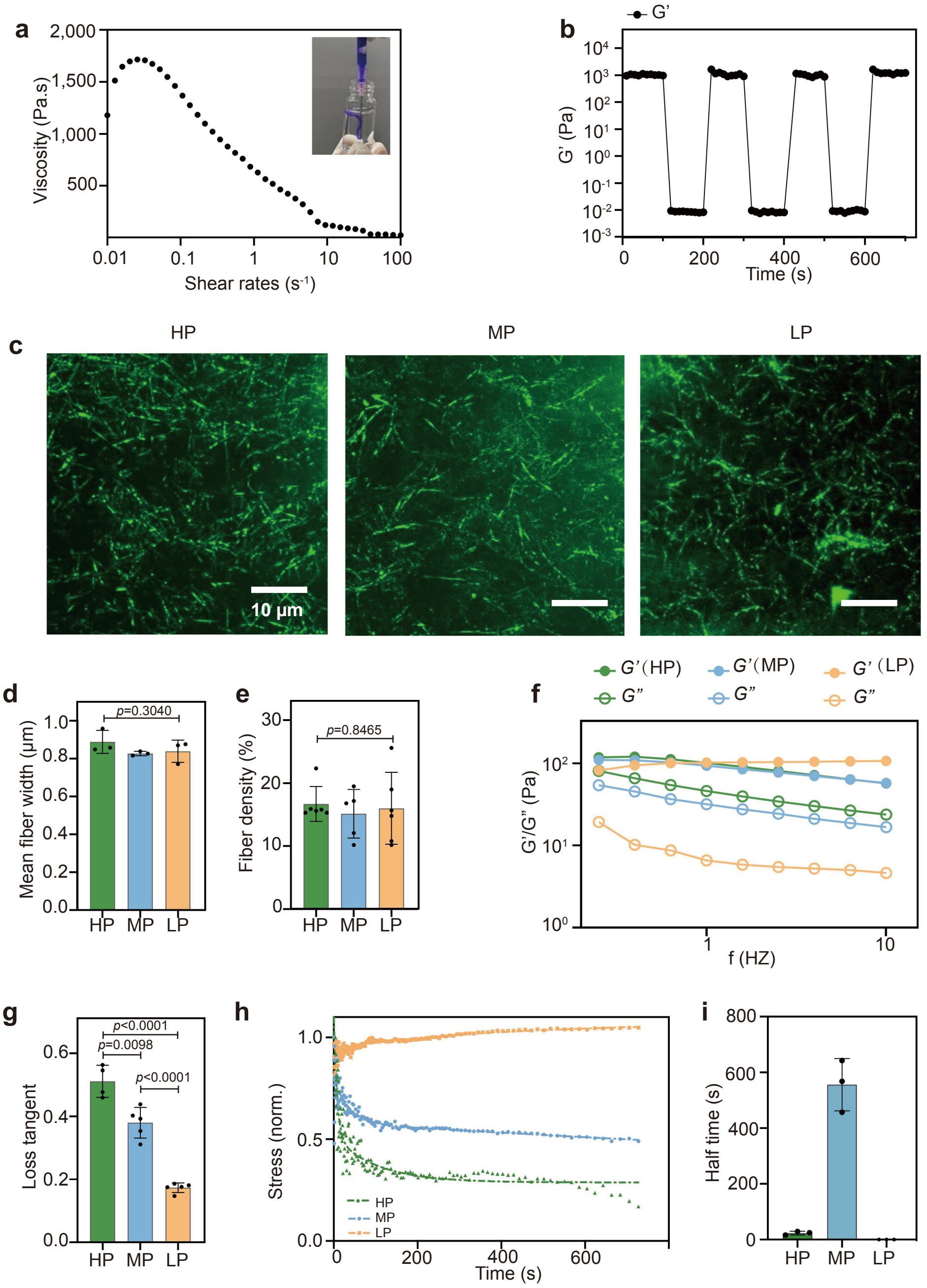

Supplementary Figure 2 | The injectability and shear-thinning of the plastic HA hydrogels, fibrous structures, and the rheological tests of the Col-HA hydrogels.

a, Viscosity and injectability of a single network of host-guest HA hydrogel, the inset shows that hydrogel is injected through a syringe. **b,** Step-strain rheological measurements of shear-thinning behaviors of the single network of host-guest HA hydrogel. **c,** The fiber structure of the HP, MP, and LP hydrogel. **d,** Mean fiber width of

the Col-HA hydrogels (n=3 samples). **e**, Fiber density of the Col-HA hydrogels (n=5 samples), Scale bar: 10 μm. **f,** Representative oscillatory frequency sweeps show *G'* and *G"* of HP, MP, and LP hydrogels. **g,** The loss tangent of *G"*/*G'* of HP, MP, and LP hydrogels (n=4 samples). **h,** Stress relaxation curves for HP, MP, and LP hydrogels with normalized stress to initial measurements. **i,** Quantification curves of timescale at which the stress is relaxed to half its original value from stress relaxation test of HP, MP, and LP hydrogels (n=3 samples). All the data are presented as mean values ± SEM. One-way analysis of variance (ANOVA) and two-tailed Student's t-tests are used to assess statistical significance.

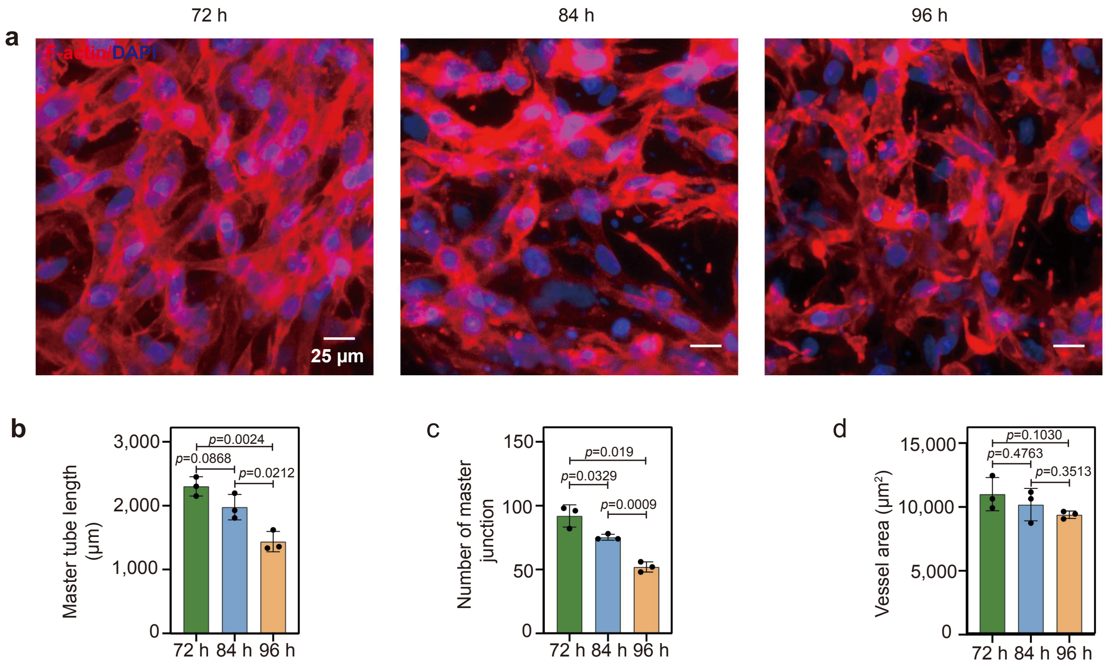

Supplementary Figure 3 | Characterization of vascular morphology of HP hydrogels after 72 h.

a, Representative confocal immunofluorescent images show morphology changes of encapsulated ECs in HP at 72 h, 84 h, and 96 h in incubation (F-actin in red and nuclei in blue). Scale bars: 25 μm. **b-d,** Quantitative analysis of vascular tube formation of 72 h, 84 h, and 96 h in HP hydrogels including **(b)** master tube length (n=3 tests), **(c)** number of master junctions (n=3 tests), **(d)** vessel area (n=3 tests). All the data are presented as mean values ± SEM. Two-tailed Student's t-tests are used to assess statistical significance.

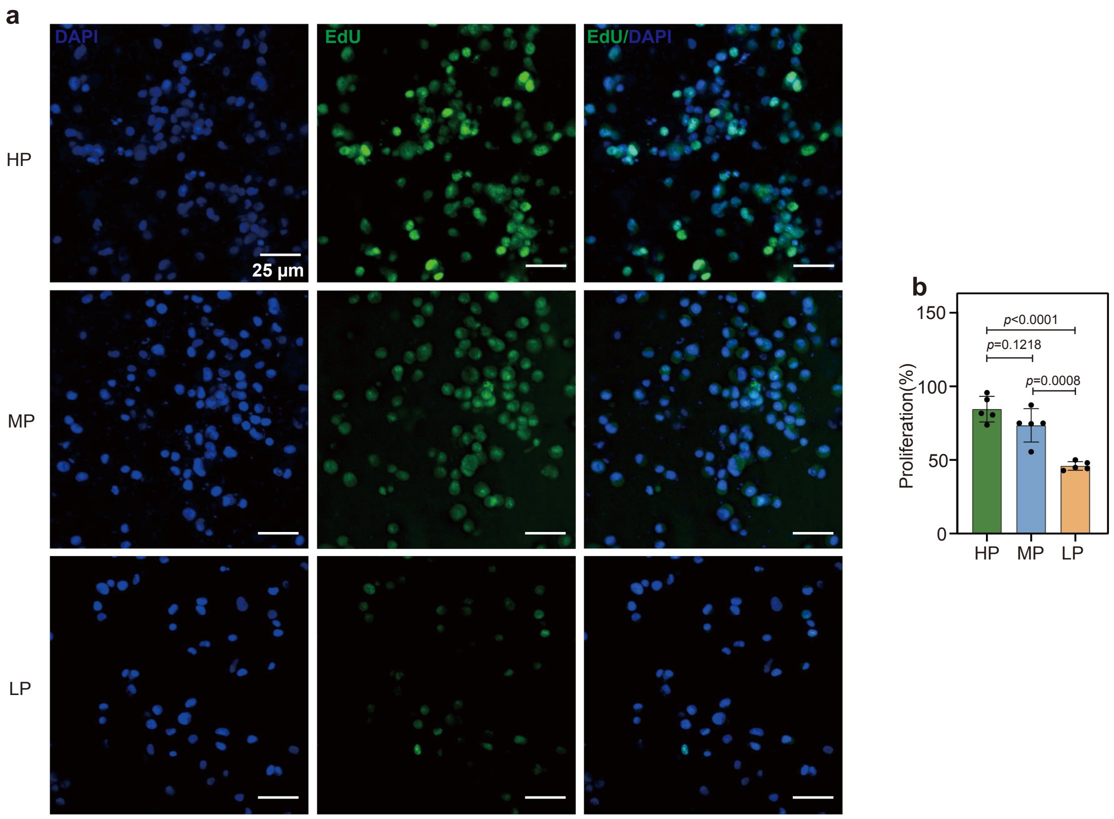

Supplementary Figure 4 | Cell proliferation ability in HP, MP, and LP hydrogels.

a, EdU assay of cells in HP, MP, and LP hydrogels. EdU-positive nuclei (labeled with Alexa Fluor 488 azide; green) and DAPI-stained nuclei of all the cells (blue) were visualized by fluorescence microscopy. Scale bars: 25 μm. **b,** Statistical graph of the EdU assay in (A) (n=5 tests). All the data are presented as mean values ± SEM and two-tailed Student's t-tests are used to assess statistical significance.

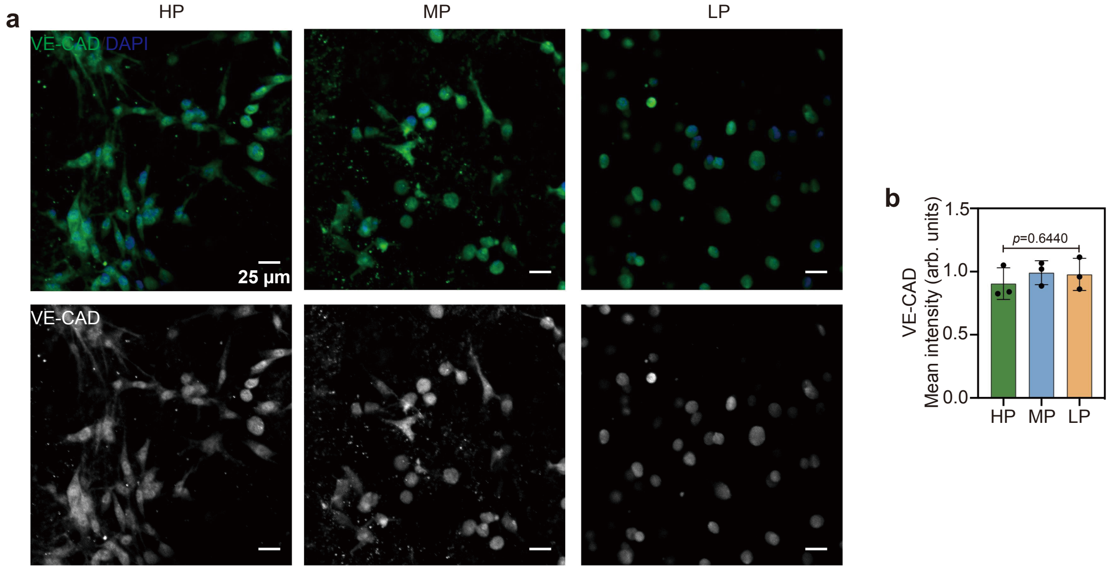

Supplementary Figure 5 | Expression of intercellular VE-CAD in HP, MP, and LP hydrogels.

a, Representative immunofluorescent images of VE-CAD of HUVECs encapsulated in HP, MP, and LP hydrogels (VE-CAD in green, nuclei in blue). Scale bars: 25 μm. **b,** Quantification of the normalized intensity of VE-CAD (n=3 tests). All the data are presented as mean values ± SEM. One-way analysis of variance (ANOVA)is used to assess statistical significance.

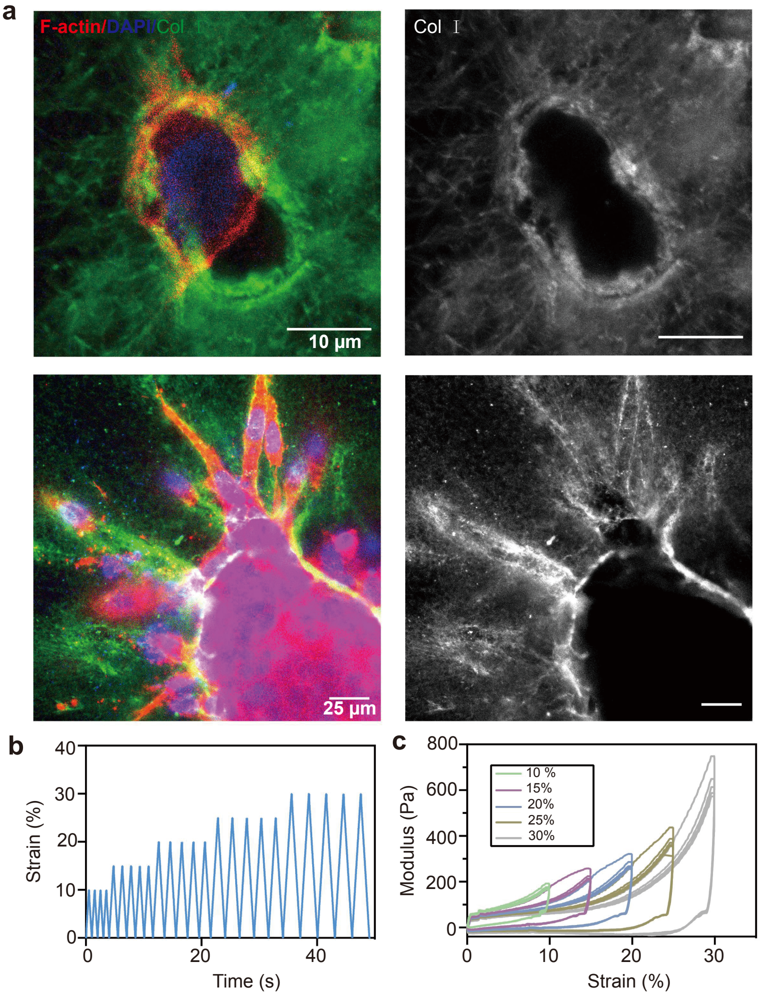

Supplementary Figure 6 | Illustrations of hydrogel network remodeling by cells.

a, High-resolution images from a confocal microscope display increased Col I density surrounding a singular cell embedded within HP hydrogel, alongside adjacent space. Scale bars: 10 μm. **b,** Depictions of an EC spheroid embedded in HP hydrogel reveal enhanced collagen density in proximity to the cell and an accompanying channel. Color keys: Col I in green, DAPI in blue, and phalloidin in red. Scale bars: 25 μm. **c,** Cyclic loading test with increasing amplitude. **d,** The cyclic stress-strain of HP hydrogel shows plastic remodeling. During cyclic loading, the strain memory effect can be observed.

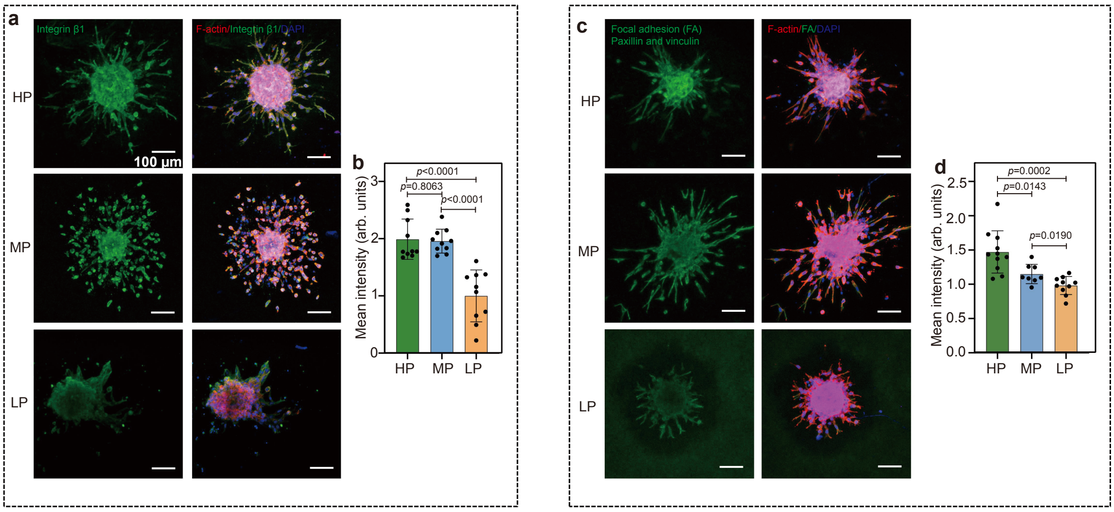

Supplementary Figure 7 | EC spheroids encapsulated in hydrogels with different plasticity.

a, b, Representative immunofluorescence images and quantification of the normalized intensity of integrin β1 of EC spheroids encapsulated in HP, MP, and LP hydrogels (F-actin in red, β1 integrin in green, nuclei in blue) (n=10 spheroids). **c, d,** Representative immunofluorescence images and quantification of the normalized intensity of FA of EC spheroids encapsulated in hydrogels of different plasticity (F-actin in red, FA in green, nuclei in blue) (from left to right n=11, 8, 10 spheroids). Scale bars: 100 μm. All the data are presented as mean values ± SEM from three independent experiments. Two-tailed Student's t-tests are used to assess statistical significance.

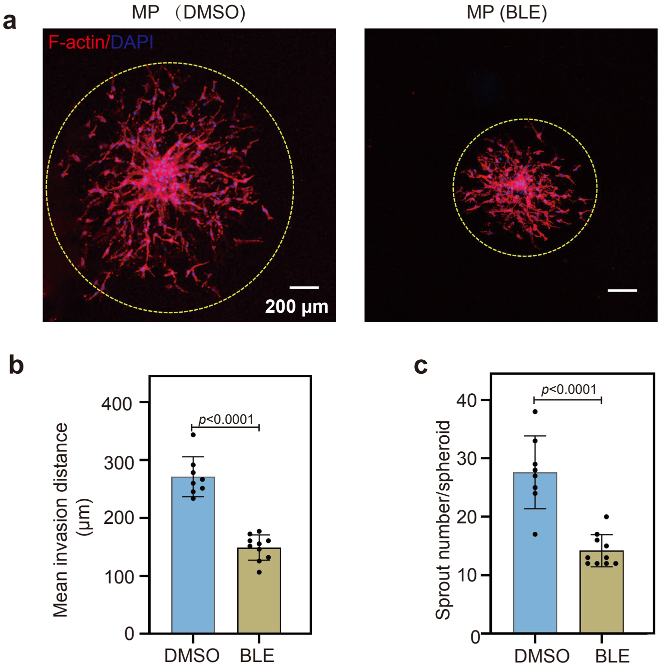

Supplementary Figure 8 | Inhibition of cell contractility weakens the sprouting and invasive ability of ECs.

a, Representative immunofluorescent images show that adding blebbistatin (BLE) inhibits the ECs sprouting in MP hydrogels. Scale bar: 200 μm. **b, c,** Quantitative analysis of the invasive capacity of EC spheroids encapsulated in MP hydrogels by using BLE inhibitors including **(b)** mean invasion distance (from left to right n=8,10 spheroids), **(c),** sprout number per spheroid (from left to right n=8, 10 spheroids). All the data are presented as mean values ± SEM from three independent experiments. Two-tailed Student's t-tests are used to assess statistical significance.

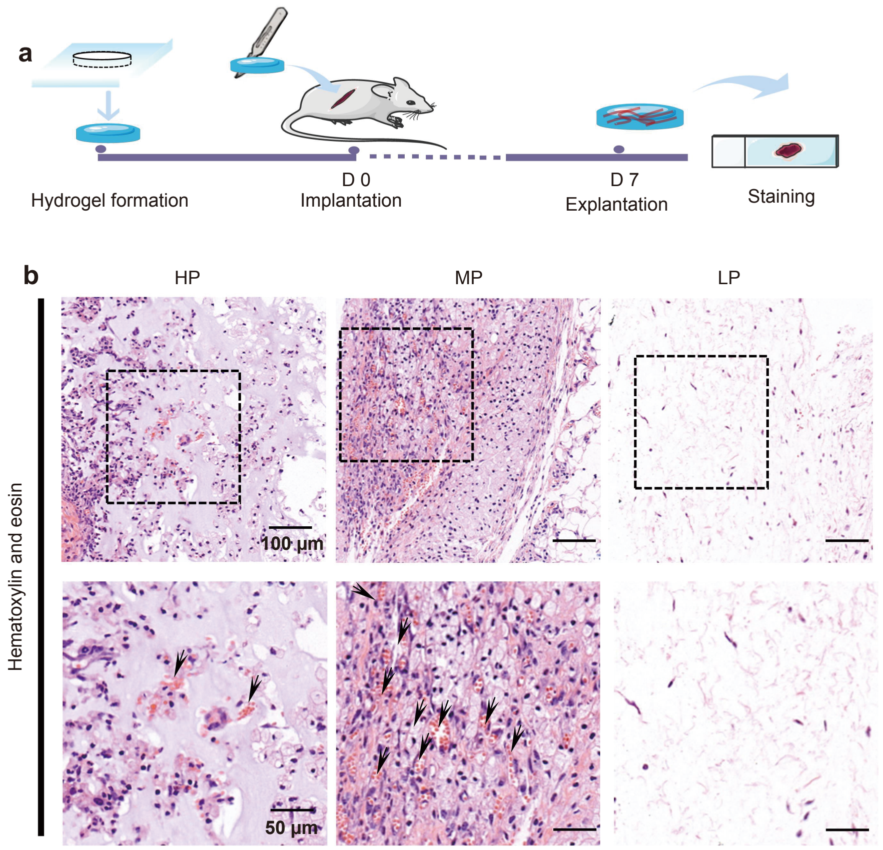

Supplementary Figure 9 | Hydrogels with different plasticity were implanted *in vivo* for angiogenic effects.

a, Schematic diagram of the hydrogel samples surgically implanted into the subcutaneous backs of mice (n = 5 for each type of hydrogel), and retrieved after 7 d.

b, Representative hematoxylin and eosin (H&E) staining of hydrogel implants on day 7. (Arrows for vessels, Scale bars: 50 and 25 μm).

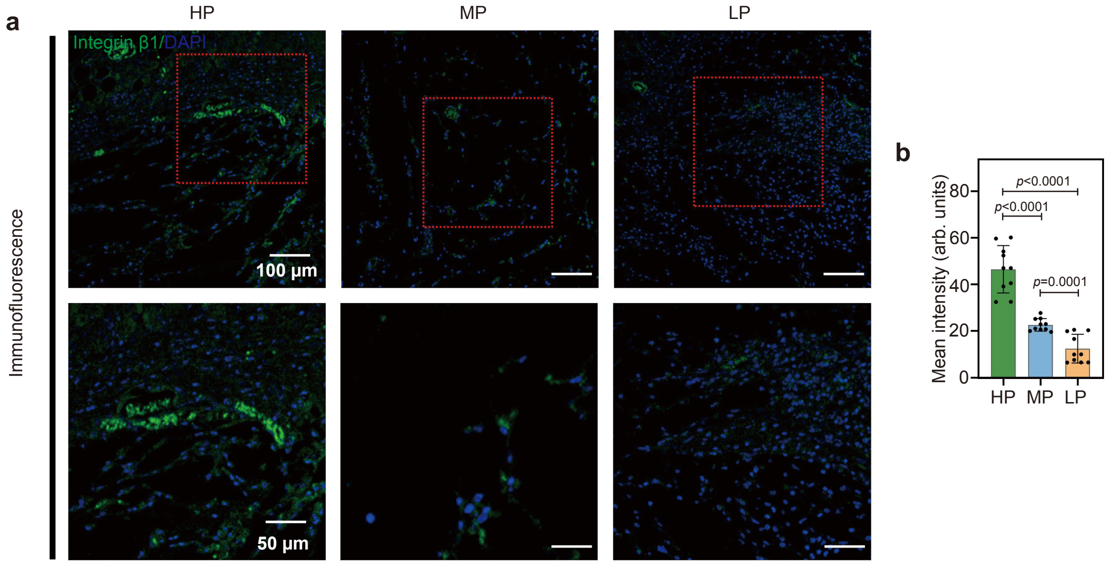

Supplementary Figure 10 | Staining of integrin β1 in extracted HP, MP, and LP hydrogel samples.

a, Representative immunofluorescent images of the hydrogel stained with integrin β1 (Integrin β1 in green, and nuclei in blue, Scale bars: 100 and 50 μm). **b,** Quantitative mean density for integrin β1 positive in hydrogels (n=10). All the data are presented as mean values ± SEM, n=5 mice with a total of 10 images were analyzed per group, Student's t-tests are used to assess statistical significance.

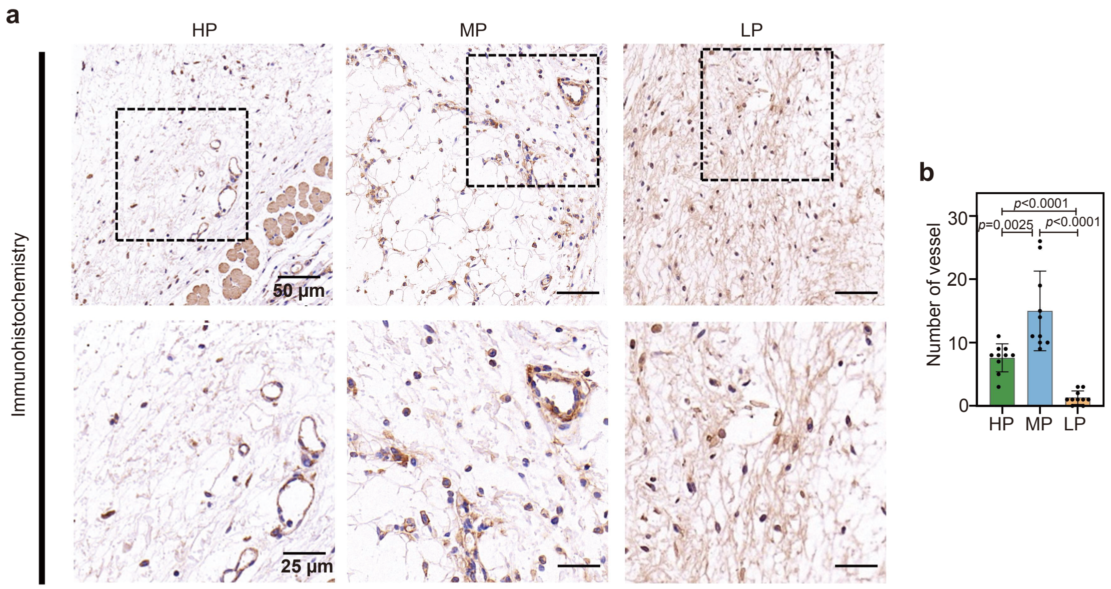

Supplementary Figure 11 | The staining of α-SMA in extracted HP, MP, and LP hydrogel samples.

a, Immunohistochemistry images of microvessels stained positive for α-SMA (Scale bars: 50 and 25 μm). **b,** Quantitative analysis of the number of vessels for α-SMA positive (n=10). All the data are presented as mean values ± SEM, n=5 mice with a total of 10 images were analyzed per group. Student's t-tests are used to assess statistical significance.
